# Performance of a simplified HEART score and HEART-GP score for evaluating chest pain in urgent primary care

**DOI:** 10.1007/s12471-020-01529-4

**Published:** 2021-01-06

**Authors:** R. E. Harskamp, M. Kleton, I. H. Smits, A. Manten, J. C. L. Himmelreich, H. C. P. M. van Weert, R. P. Rietveld, W. A. M. Lucassen

**Affiliations:** 1 Department of General Practice, Amsterdam UMC, University of Amsterdam, Amsterdam Cardiovascular Sciences and Amsterdam Public Health Research Institutes, Academic Medical Centre, Amsterdam, The Netherlands; 2 Holendrecht Medical Center, Amsterdam, The Netherlands; 3 Huisartsenorganisatie Noord-Kennemerland, Alkmaar, The Netherlands; 4 Huisartsen Centrumwaard, Heerhugowaard, The Netherlands; 5 Huisartsen Risdam, Zwaag, The Netherlands

**Keywords:** Chest pain, Primary care, Major adverse cardiac events, HEART score

## Abstract

**Background:**

Chest pain is a common symptom in urgent primary care. The distinction between urgent and non-urgent causes can be challenging. A modified version of the HEART score, in which troponin is omitted (‘simplified HEART’) or replaced by the so-called ‘sense of alarm’ (HEART-GP), may aid in risk stratification.

**Method:**

This study involved a retrospective, observational cohort of consecutive patients evaluated for chest pain at a large-scale, out-of-hours, regional primary care facility in the Netherlands, with 6‑week follow-up for major adverse cardiac events (MACEs). The outcome of interest is diagnostic accuracy, including positive predictive value (PPV) and negative predictive value (NPV).

**Results:**

We included 664 patients; MACEs occurred in 4.8% (*n* = 32). For  simplified HEART and HEART-GP, we found C‑statistics of 0.86 (95% confidence interval (CI) 0.80–0.91) and 0.90 (95% CI 0.85–0.95), respectively. Optimal diagnostic accuracy was found for a simplified HEART score ≥2 (PPV 9%, NPV 99.7%), HEART-GP score ≥3 (PPV 11%, NPV 99.7%) and HEART-GP score ≥4 (PPV 16%, NPV 99.4%). Physicians referred 157 patients (23.6%) and missed 6 MACEs. A simplified HEART score ≥2 would have picked up 5 cases, at the expense of 332 referrals (50.0%, *p* < 0.001). A HEART-GP score of ≥3 and ≥4 would have detected 5 and 3 MACEs and led to 293 (44.1%, *p* < 0.001) and 186 (28.0%, *p* = 0.18) referrals, respectively.

**Conclusion:**

HEART-score modifications including the physicians’ ‘sense of alarm’ may be used as a risk stratification tool for chest pain in primary care in the absence of routine access to troponin assays. Further validation is warranted.

**Supplementary Information:**

The online version of this article (10.1007/s12471-020-01529-4) contains supplementary material, which is available to authorized users.

## What’s new?

- A simplified HEART score based on the elements history, electrocardiogram, age, and risk factors may present a safe risk stratification tool in urgent primary care.
- A modified HEART score (HEART-GP) in which the physician’s own gut feeling (‘sense of alarm’) is included may further improve accuracy and, particularly, efficiency.
- Both scores represent a safe, albeit less efficient, risk stratification tool when compared with unaided clinical judgement.

## Introduction

Chest pain is a common reason for consulting general practitioners (GPs). Approximately 1–4% of all new episodes are related to chest pain [1–5]. The principle task for GPs lies in differentiating urgent (but uncommon) causes of chest pain from the less urgent underlying conditions of the majority of patients [2, 6]. To make this differentiation GPs mainly depend on prior experience, past medical history, and careful history taking, at times a rather tricky endeavour [7, 8]. So what can GPs do to optimise risk stratification of patients with chest pain? One possibility is to explore the feasibility of using a decision support tool, such as the ‘HEART’ score [9–12]. While the HEART score is a robust risk stratification tool in the emergency department (ED), its performance is unknown in (unselected) primary care populations, a setting where quantitative troponin assays are not routinely available. Furthermore, the HEART score does not take into account a physician’s gut feeling (hereafter referred to as ‘sense of alarm’), which is often the trigger for GPs to refer a patient [13, 14]. In this study we therefore evaluated the diagnostic performance of a simplified HEART score (omitting troponin) and HEART-GP score (replacing troponin with sense of alarm) to risk-stratify patients with chest pain in urgent primary care.

## Methods

We reported this diagnostic accuracy study in accordance with the Standards for Reporting of Diagnostic Accuracy Studies (STARD) 2015 statement [15]. This study protocol was evaluated by our institution’s Medical Ethical Review Committee (TRACE) [16]. All patients were informed by mail of the conduct of this study and were provided with the opportunity to opt out of sharing data for this study [16].

### Study design

This study involved a retrospective, observational cohort of consecutive patients (≥18 years) evaluated for chest pain at a large regional primary care facility in Alkmaar, the Netherlands in 2017. The facility is responsible for out-of-office-hours urgent primary care for 245,000 inhabitants. Evaluation involved anamnesis, physical examination, and 12-lead electrocardiogram (ECG), at the discretion of the treating physician. Follow-up information was obtained from electronic health records from the GP, and outpatient, admission or discharge notes from the ED/hospital.

### Simplified HEART and HEART-GP scores

The simplified HEART score consists of: history, ECG, age, and risk factors. For the HEART-GP score a fifth element is added, which is based on the GP’s sense of alarm, as shown in Tab. 1. For interpretation of the history element, we relied on the approach previously reported by Mahler et al. [11, 12]. In their study the history element depends on balancing low- and high-risk features. We presumed the absence of a high-risk symptom when such a feature was not recorded in the electronic health records by the treating physician.

**Table 1:** Elements of the HEART-GP score and points assigned

Elements	Points
*History (symptoms)*^a^	
Slightly suspicious	0
Moderately suspicious	1
Highly suspicious	2
*Electrocardiographic findings*^b^	
Normal	0
Non-specific repolarisation disturbance	1
Significant ST depression	2
*Age*	
<45 years	0
45–64 years	1
≥65 years	2
*Risk factors*^c^	
None	0
1–2	1
≥3 or history of atherosclerosis	2
*Sense of alarm*^d^	
Low	0
Moderate	1
High	2

^a^ History is based on high-risk and low-risk features. High-risk features include: pain in middle or on left side of chest, pressure-type pain/tightness, worse pain on exertion, pain relieved by nitroglycerin, radiation of pain to arms/jaw/neck, nausea or vomiting and diaphoresis. Low-risk features include: pinpoint/well-localised pain and sharp pain. The presence of 4 high-risk features led to assigning 2 points, 2–3 high-risk features to 1 point, and fewer high-risk features to zero points. Each low-risk feature neutralised a high-risk feature [11, 12]

^b^Non-specific repolarisation disturbance consists of: repolarisation abnormalities, non-specific T‑wave changes, non-specific ST changes, bundle branch blocks, pacemaker rhythms, left ventricular hypertrophy, early repolarisation, digoxin effect

^c^Risk factors for coronary artery disease include: family history of atherosclerotic disease, diabetes mellitus (currently treated), hypertension, hypercholesterolaemia, smoking in the past 90 days or obesity (body mass index ≥30 kg/m^2^). A history of atherosclerosis involves: history of myocardial infarction, transient ischaemic attack, cerebrovascular accident, peripheral artery disease, previous percutaneous coronary intervention or coronary artery bypass graft

^d^The component ‘sense of alarm’ triggers the GP’s reaction based on a gut feeling. Low sense of alarm leads to follow-up with own GP during office hours or no recommendation for follow-up. Moderate sense of alarm results in non-urgent referral or telephone consultation with specialist. High sense of alarm triggers immediate referral to the emergency department and/or ambulance activation

### Major adverse cardiac events

The primary outcome of interest is the occurrence of a major adverse cardiac event (MACE) occurring within 6 weeks of initial contact with the GP. MACE is defined as a composite consisting of death from any cause, acute coronary syndrome (ACS), or coronary revascularisation.

### Data collection

Study personnel visited the out-of-office-hours primary care facility as well as the affiliated primary care practices in the Alkmaar region to collect baseline and follow-up information from electronic health records. Baseline data included sex, age, medical history, and use of relevant medications. Data were collected and processed using a secure, web-based, electronic data capture platform (Castor EDC, Amsterdam, The Netherlands). Further information on the methodology used for data collection can be found in a methodology paper published previously by our group [16].

### Statistical analysis

We expressed diagnostic accuracy for the simplified HEART and HEART-GP scores for detecting 6‑week MACEs at various thresholds as sensitivity, specificity, accuracy, positive and negative predictive values (PPV, NPV), with 95% confidence intervals (CI). We displayed the overall discriminatory properties using C‑statistics.

## Results

### Baseline characteristics

During the study period, a total of 770 patients were evaluated by a GP for chest pain. We had to exclude data from 83 of these patients who objected to sharing medical data for research purposes (in the wake of the introduction of new European data protection regulations). Of the remaining patients, we could not obtain follow-up information on 23 (3.3%), which left us with a study population of 664 patients. The baseline characteristics of these patients are shown in Tab. 2. Overall, the median age was 48 years, and 56.9% were female. Risk factors for cardiovascular disease were common (39.8%), of which hypertension (25.5%) had the highest prevalence. Symptom characteristics were also different, with MACE cases more often having heavy/pressure-type chest pain with radiation, nausea and diaphoresis, and less often localised pain that is reproducible by palpation.

**Table 2:** Baseline characteristics of study population

	Total (*n* = 664)	MACEs (*n* = 32)	No MACEs (*n* = 632)
*Age in years (median, 25th–75th percentiles)*	48 (32–67)	72 (68–79)	46 (31–66)
*Male*	286 (43.1%)	20 (62.5%)	266 (42.1%)
*Cardiovascular risk factors*			
Smoking (current)	119/439 (27.1%)	2/23 (8.7%)	117/416 (28.1%)
Hypertension	169 (25.5%)	18 (56.3%)	151 (23.9%)
Hypercholesterolaemia	83 (12.5%)	8 (25.0%)	75 (11.9%)
Diabetes mellitus	55 (8.3%)	4 (6.3%)	51 (8.1%)
Family history of atherosclerotic disease	54 (8.1%)	2 (6.3%)	52 (8.2%)
Obesity	12 (1.8%)	1 (3.1%)	11 (1.7%)
*History of cardiovascular disease*			
Myocardial infarction	43 (6.5%)	6 (18.8%)	37 (5.9%)
CVA/TIA	34 (5.1%)	4 (12.5%)	30 (4.7%)
PAD	10 (1.5%)	1 (3.1%)	9 (1.4%)
PCI	44 (6.6%)	7 (21.9%)	37 (5.9%)
CABG	12 (1.8%)	1 (3.1%)	11 (1.7%)
*Use of cardiovascular medications*			
Platelet aggregation inhibitor	27 (4.1%)	1 (3.1%)	26 (4.1%)
Salicylates	69 (10.4%)	8 (25.0%)	61 (9.7%)
Statins	119 (17.9%)	9 (28.1%)	110 (17.4%)
Beta-blockers	118 (17.8%)	10 (31.3%)	108 (17.1%)
ACE inhibitors/ARBs	118 (17.8%)	18 (56.3%)	100 (15.8%)
Vitamin K antagonist	48 (7.2%)	4 (12.5%)	44 (7.0%)
NOACs	19 (2.9%)	1 (3.1%)	18 (2.8%)
Nitrates	46 (6.9%)	7 (21.9%)	39 (6.2%)
*Chest pain duration*			
<1 h	13 (2.0%)	0 (0.0%)	13 (2.1%)
1–24 h	317 (47.7%)	16 (50.0%)	301 (47.6%)
>24 h	240 (36.1%)	14 (43.8%)	226 (35.8%)
Not specified	94 (14.2%)	2 (6.3%)	92 (14.6%)
*Chest pain presentation*			
Pain in middle or on left side of chest	302 (45.5%)	14 (43.8%)	288 (45.6%)
Heavy/pressure/tightness	204 (30.7%)	17 (53.1%)	187 (29.6%)
Worse pain on exertion	70 (10.5%)	3 (9.4%)	67 (10.6%)
Pain relieved by nitroglycerin	27 (4.1%)	3 (9.4%)	24 (3.8%)
Radiation of pain to arms/jaw/neck	128 (19.3%)	13 (40.6%)	115 (18.2%)
Nausea or vomiting	85 (12.8%)	6 (18.8%)	79 (12.5%)
Lightheadedness	73 (11.0%)	2 (6.3%)	71 (11.2%)
Diaphoresis	61 (9.2%)	7 (21.9%)	54 (8.5%)
*Other relevant symptoms*			
Dyspnoea	169 (24.5%)	8 (25.0%)	161 (25.5%)
Cough	80 (12.0%)	0 (0.0%)	80 (12.7%)
*Physical examination*			
Localised pain	152 (22.9%)	1 (3.1%)	151 (23.9%)
Pain reproducible with palpation	257 (38.7%)	3 (9.4%)	254 (40.2%)
Heart rate (bpm)	80 (70–90)	80 (61–95)	80 (70–90)
Systolic blood pressure (mm Hg)	136 (120–150)	150 (140–175)	135 (120–150)
Diastolic blood pressure (mm Hg)	80 (75–90)	80 (78–100)	80 (75–90)
Pulse oximeter, saturation (%)	98 (97–99)	97 (95–98)	98 (97–99)
Normal heart sounds	378/389 (97.2%)	18/21 (85.7%)	371/379 (97.9%)
Normal pulmonary sounds	477/527 (90.5%)	20/22 (90.9%)	457/505 (90.5%)
Fever	17 (2.6%)	1 (3.1%)	16 (2.5%)

*MACEs* major adverse cardiac events, *CVA* cerebrovascular accident, *TIA* transient ischaemic attack, *PAD* peripheral artery disease, *PCI* percutaneous coronary intervention, *CABG* coronary artery bypass graft, *ACE* angiotensin converting enzyme, *ARBs* angiotensin II receptor blockers, *NOACs* novel oral anticoagulants

### Clinical outcomes

**Fig. 1:**
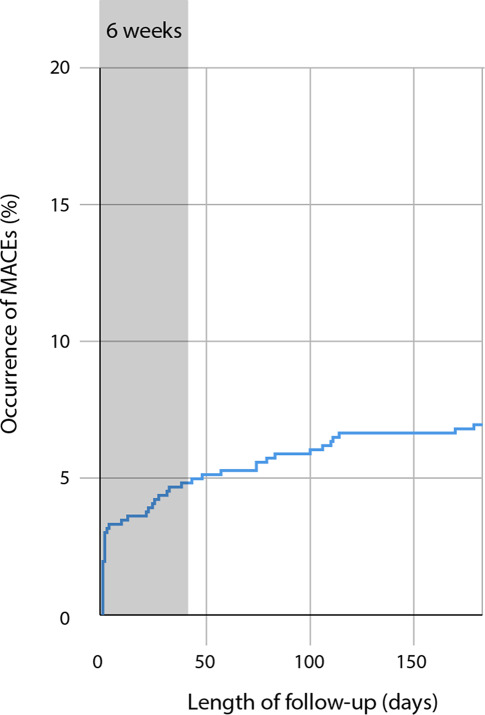
Occurrence of major adverse cardiac events (*MACEs*, %) in the study population over a 6-month time window

A total of 32 (4.8%) patients suffered a MACE within the first 6 weeks after consultation (Fig. 1). Of those 6 died (5 from cardiovascular causes), 6 patients had an ST-segment elevation myocardial infarction, 14 non-ST-segment elevation myocardial infarction, 4 unstable angina, and 2 patients underwent coronary revascularisation. Apart from MACEs, there were also 10 cases of heart failure, 7 cases of pulmonary embolism, and 1 patient with a (non-fatal) aortic dissection who underwent supracoronary aortic replacement surgery. A complete list of events can be found in the Electronic Supplementary Material (Table S1).

### Physician performance

After initial evaluation, GPs urgently referred a total of 157 (23.6%) patients to the (cardiac) ED, 74 by ambulance and 83 with self-transportation. Of those, a total of 26 had a MACE within 6 weeks (PPV 16.6%, 95% CI 13.7–19.9%). A total of 6 patients were not referred but still had a MACE within 6 weeks (NPV 98.8%, 95% CI 97.6–99.4%). The sensitivity and specificity were 81.3%, 95% CI 63.6–92.8% and 79.3%, 95% CI 75.9–82.4%, respectively.

### Performance of the simplified HEART and HEART-GP scores

**Fig. 2:**
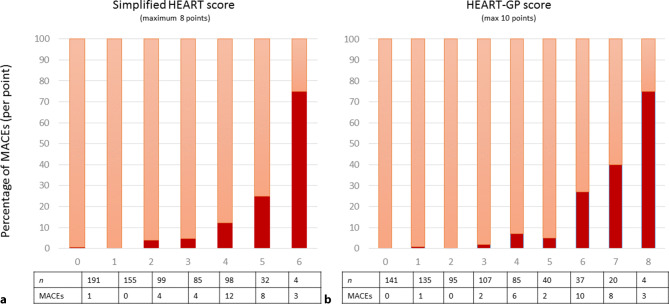
Percentage of major adverse cardiac events (*MACEs*) per point of a simplified HEART (**a**) and HEART-GP (**b**) score

**Fig. 3:**
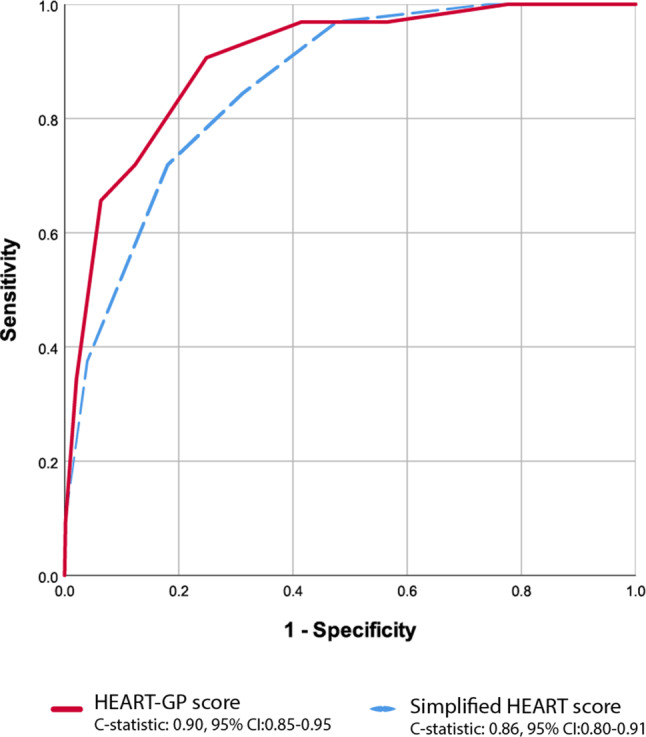
Summary of receiver operating characteristic curve of specificity and sensitivity of a simplified HEART and HEART-GP score

The distribution of the simplified HEART and HEART-GP scores and the occurrence of MACEs can be found in Fig. 2. Overall, the occurrence of MACEs was rare in those patients with a low score on the simplified HEART (1/346 = 0.29% for score ≤1) or HEART-GP (1/371 = 0.27% for score ≤2), and increased to 75% in those with the highest documented simplified HEART score (=6/8 points) or HEART-GP score (=8/10 points), respectively. When assessing the individual components, patient history, ECG abnormalities, age, and risk factors were all associated with MACEs (Electronic Supplementary Material, Table S2). As shown in Fig. 3, the simplified HEART and HEART-GP scores had C‑statistics of 0.86, 95% CI 0.80–0.91 and 0.90, 95% CI 0.85–0.95, respectively. The diagnostic performance of the simplified HEART and HEART-GP scores at various thresholds (1–5) is summarised in Tab. 3. In short, the NPV was at or above 99% when applying referral thresholds of 3 points (or lower) for the simplified HEART score and 4 points (or lower) for the HEART-GP score, respectively. The number of false-negative cases remained low (≤5 cases) when applying a threshold of ≤3 points for the simplified HEART score, or ≤4 points for the HEART-GP score.

**Table 3:** Diagnostic properties of the simplified HEART and HEART-GP scores at different thresholds (scores of 1–5)

	Threshold (score)	Referred (*n*)	MACEs (missed/*n*)	Sensitivity (%, 95th CI)	Specificity (%, 95th CI)	PPV (%, 95th CI)	NPV (%, 95th CI)	Accuracy (%, 95th CI)
Simplified HEART	≥5	37	20/32	38 (21–56)	96 (94–97)	32 (21–46)	97 (96–98)	93 (91–95)
	≥4	137	9/32	72 (53–86)	82 (79–85)	17 (13–21)	98 (97–99)	81 (78–84)
	≥3	224	5/32	84 (67–95)	70 (66–73)	12 (10–15)	99 (98–100)^a^	70 (66–73)
	≥2	332	1/32	97 (84–100)	52 (48–56)	9 (9–10)	100 (98–100)^b^	54 (51–58)
	≥1	501	0/32	100 (89–100)	26 (22–29)	6 (6–7)	100 (–)	29 (26–33)
HEART-GP	≥5	110	9/32	72 (53–86)	88 (85–90)	23 (18–28)	98 (97–99)	87 (84–89)
	≥4	186	3/32	91 (75–98)	75 (72–78)	16 (13–18)	99 (98–100)^c^	76 (72–79)
	≥3	293	1/32	97 (84–100)	58 (55–62)	11 (10–12)	100 (98–100)^d^	60 (57–64)
	≥2	388	1/32	97 (84–100)	44 (40–47)	8 (7–9)	100 (98–100)^e^	46 (42–50)
	≥1	523	0/32	100 (89–100)	22 (19–26)	6 (6–6)	100 (–)	26 (23–30)

*MACEs *major adverse cardiac events, *CI* confidence interval, *PPV* positive predictive value, *NPV* negative predictive value

^a^98.9% (97.5–99.5)

^b^99.7% (98.0–99.96)

^c^99.4% (98.2–99.8)

^d^99.7% (98.2–99.96)

^e^99.6% (97.6–99.95)

### Simplified HEART score and HEART-GP score versus physician assessment

We found a lower number of missed MACEs when using a simplified HEART score of ≥2 points (1 missed case, 0.15%) or a HEART-GP score of ≥3 or ≥4 points (1 (0.15%) or 3 (0.45%) missed cases) as a referral threshold, instead of unassisted physician assessment (6 missed cases (=0.90%)). This improved safety comes at the expense of additional referrals. For a simplified HEART score of ≥2 points this would lead to 175 (332 vs 157, 50.0% vs 23.6%, *p* < 0.001) additional referrals when compared with physician assessment. For the HEART-GP score, a threshold of ≥3 points would lead to a total of 136 additional patient referrals (293 vs 157, 44.1% vs 23.6%, *p* < 0.001). For a HEART-GP score of ≥4 points there would be 29 additional referrals (186 vs 157, 28.0% vs 23.6%, *p* = 0.08). Finally, when comparing unaided physician performance with a high-threshold referral strategy, such as a HEART-GP score of ≥5 points, we would see fewer referrals (110 vs 157, *p* < 0.001), but also more missed cases (9 vs 6).

## Discussion

Chest pain is a common symptom and often presents a clinical challenge for GPs, particularly in the setting of out-of-hours service. In the (cardiac) emergency ward a number of risk stratification tools have been developed, of which the HEART score is the most commonly used, due to its ease-of-use and reliability [9–11]. In primary care, a stratification tool, such as the HEART score, is currently lacking. Seen in this light, the findings of our study are of interest, as they illustrate that a simplified version of this score relying on history, ECG, age, risk factors, and the physician’s sense of alarm may be able to improve decision making in primary care. In our study, we found that the simplified HEART score and the HEART-GP score both had good diagnostic properties (C-statistic of >0.85, and NPV exceeding 99% at cut-off values of ≥2 or ≥3/4, respectively). Compared with physician assessment, we found that the simplified HEART score of ≥2 points and HEART-GP score of ≥3/4 points could further improve safety. We found that this increased safety comes at the expense of referring (almost) half instead of a quarter of the evaluated patients with chest pain. In this regard, the inclusion of the physician’s sense of alarm (HEART-GP score) performed better than the simplified HEART score.

### Strengths and limitations

Our study involved the clinical presentation and clinical course of consecutive patients with chest pain in urgent primary care, which curtails the risk of selection bias. The study involved a relatively large number of patients and was conducted in a large-scale urgent primary care centre, involving over a hundred GPs, and is therefore likely a representative sample. Prior studies have found that particularly the history element is prone to subjective interpretation. To minimise this heterogeneity, we applied a rigorous approach in which we scored high- and low-risk features as previously described by Mahler et al. [11]. These assessments were made by experienced investigators who were blinded as to the final diagnosis and/or outcome. The limitations of the study are as follows: the study was retrospective in nature, and we presumed absence of a symptom when a symptom or other element was not recorded by the treating physician. The number of MACEs is limited, and we can therefore not rule out a certain degree of imprecision in regard to the diagnostic performance of the studied risk scores. Another limitation is selective clinical work-up and follow-up, which may have led to verification bias. Finally, a mentionable number of GPs refused to provide follow-up data of their patients because of the ‘opt-out-plus’ design of the study, or expressed liability concerns due to the recently implemented European data protection regulations.

### Clinical perspective: playing the odds

Previously, our group conducted a survey among ≈300 GPs to establish what they would perceive as an acceptable rate for missed MACEs among patients who present with acute-onset chest pain [8]. Most GPs would be willing to accept missing 0.5–2.5% of cases, while at the same time keeping the referral threshold to a maximum of 50 ‘unnecessary’ referrals for each ACS case. Based on our study, the simplified HEART score would likely not be of added value. A threshold of ≥2 points would result in too many referrals, whereas a threshold of ≥3 points would not lead to a substantial reduction in the number of missed cases. The HEART-GP score seems more promising, either using a threshold of ≥3 points (higher referral rate, but very low rate of missed cases), or ≥4 points (29 additional referrals and 3 fewer missed MACEs).

### Prior studies to establish clinical decision rules in primary care

A number of studies have been conducted to construct a clinical decision rule over the past three decades. In the late 1990s Grijseels et al. developed a decision aid for ruling out ACS in general practice [17]. Risk assessment in this aid was based on ECG parameters and high-risk features (male sex, past medical history of coronary artery disease) and symptoms (presence of radiation of pain and/or nausea/sweating). This score was recalibrated by Bruins Slot et al. in 2011 [18]. These studies showed mediocre discriminatory properties (C-statistic 0.66–0.72), and unaided clinical judgement provided a better overall fit (C-statistic of 0.75), with poor agreement in risk estimation (in half of cases) [6, 17, 18]. Recently, a 2-week flash-mob study was performed among Dutch GPs in which the Marburg Heart Score was evaluated for its properties for ruling out ACS in patients referred for suspected ACS [19]. Overall, the diagnostic properties in terms of predictive values of the Marburg Heart Score, as for the other risk assessment tools, were not superior to unaided GP assessment.

### Future directions: point-of-care troponin

In order to uncover the full potential of the HEART score, or other risk scores, the availability of a reliable point-of-care (POC) troponin test is pivotal [6]. In the pre-hospital (ambulance) setting the use of troponin resulted in an improved performance of the HEART score (C-statistic of 0.74 vs 0.65) [20]. The ambulance-based ATTICA trial is now evaluating whether patients with a low HEART score (including troponin) could be safely deferred to primary care [21]. An urgent primary care study that evaluated the HEART score (URGENT) was terminated prematurely, as the POC troponin was retracted (and sold) by the manufacturer [22]. Overall, 37 cases could be analysed, of which 10 were referred (4 cases of ACS), and 1 case of ACS was missed (among 27 non-referred patients). The missed case was the result of a breach in protocol. Seen in this light, the findings of this pilot study are promising, and future efforts to evaluate the HEART score should be encouraged when a reliable, time-efficient, POC troponin test becomes available. Based on the findings of our study, the HEART score should perhaps be modified to also include the GP’s sense of alarm.

## Conclusion

Modified versions of the HEART score in which troponin is omitted may be used as a risk stratification tool for chest pain in urgent primary care settings. Our findings suggest safety may be improved in terms of detecting MACEs when compared with unaided clinical judgement. Furthermore, including the physician’s sense of alarm as part of the HEART score may also result in improved efficiency. Future studies are warranted to confirm our initial findings, preferably augmented with troponin, before considering implementation in urgent primary care.

## Supplementary Information

Detailed description of major adverse cardiovascular events and cases/total patients for each element of simplified HEART and HEART-GP
