# Supplementary material for: Performance of a simplified HEART score and HEART-GP score for evaluating chest pain in urgent primary care
Source: Neth Heart J. 2021 Jan 6;29(6):338–47. doi: 10.1007/s12471-020-01529-4 (PMC8160073; doi:10.1007/s12471-020-01529-4)
Supplement: Supplementary file 1 — Detailed description of major adverse cardiovascular events and cases/total patients for each element of simplified HEART and HEART-GP [file 12471_2020_1529_MOESM1_ESM.pdf]

SUPPLEMENTAL DATA

Manuscript:
Harskamp et al. Performance of simplified versions of the HEART score for evaluating chest pain in urgent primary care

Supplemental table S1. Major adverse cardiac/cardiovascular events within 6 weeks of index presentation

Case	Description	MACVE	MACE	Death	ACS	Revasc	Other
1	NSTEMI → HF → death	1	1	CV	NSTEMI		HF
2	NSTEMI and HF	1	1		NSTEMI		HF
3	IAP	1	1		IAP		
4	NSTEMI + PCI	1	1		NSTEMI	PCI	
5	STEMI + pPCI	1	1		STEMI	PCI	
6	NSTEMI + PCI	1	1		NSTEMI	PCI	
7	NSTEMI + PCI	1	1		NSTEMI	PCI	
8	NSTEMI	1	1		NSTEMI		
9	IAP	1	1		IAP		
10	NSTEMI	1	1		NSTEMI		
11	IAP	1	1		IAP		
12	NSTEMI + PCI	1	1		NSTEMI	PCI	
13	STEMI + pPCI	1	1		STEMI	PCI	
14	IAP	1	1		IAP		
15	Type A dissection	1					Aorta dis
16	HF + AF	1					HF
17	HF	1					HF
18	PE	1					PE
19	PE	1					PE
20	PE	1					PE
21	PE → death	1	1	CV			PE
22	NSTEMI	1	1		NSTEMI		
23	NSTEMI + PCI	1	1		NSTEMI	PCI	
24	NSTEMI + PCI	1	1		NSTEMI	PCI	
25	NSTEMI + PCI	1	1		NSTEMI	PCI	
26	NSTEMI + PCI	1	1		NSTEMI	PCI	
27	HF	1					HF
28	PE	1					PE
29	PE	1					PE
30	Cardiac arrest	1	1	CV			
31	HF + AF	1					HF
32	Cardiac arrest	1	1	CV			
33	STEMI + PCI	1	1		STEMI	PCI	
34	HF + AF + PCI	1	1			PCI	HF
35	HF	1					HF
36	PE	1					PE
37	Non CV death	1	1	Non CV			
38	HF	1					HF
39	HF → CV death	1	1	CV			
40	STEMI + pPCI	1	1		STEMI	PCI	
41	STEMI + pPCI	1	1		STEMI	PCI	
42	CABG	1	1			CABG	
43	STEMI + pPCI	1	1		STEMI	PCI	
44	NSTEMI + PCI	1	1		NSTEMI	PCI	
45	PCI	1	1			PCI	
46	HF	1					HF
**Total**		N=46	N=32				
	**CV death**			N=5			
	**Non CV death**			N=1			
	**STEMI**				N=6		
	**NSTEMI/IAP**				N=14/n=4		
	**PCI/CABG (non-ACS)**					N=1/n=1	
	**Aortic dissection**						N=1
	**Pulmonary embolism**						N=7
	**Heart failure**						N=10

Table S2. Patients with a MACE endpoint (within 6 weeks) for each element of the simplified HEART score as well as the physician's sense of alarm (HEART-GP)

	0 points	*1 points*	*2 points*	*P-value*
History	13/483 (2.7%)	14/159 (8.8%)	5/22 (22.7%)	<0.001
ECG	26/646 (4.0%)	2/12 (15.3%)	4/6 (80.0%)	<0.001
Age	1/307 (0.3%)	6/166 (3.6%)	25/191 (13.1%)	<0.001
Risk factors	6/312 (1.9%)	13/240 (5.4%)	13/112 (11.6%)	<0.001
Trigger PCP	5/479 (1.0%)	1/28 (3.6%)	26/157 (16.6%)	<0.001
